# Connectivity Analysis of Cognitive Radio Ad-Hoc Networks with Multi-Pair Primary Networks

**DOI:** 10.3390/s19030565

**Published:** 2019-01-29

**Authors:** Le The Dung, Seong-Gon Choi

**Affiliations:** Department of Radio and Communication Engineering, Chungbuk National University, Cheongju City, Chungbuk 28644, Korea; dung.t.le@ieee.org

**Keywords:** cognitive radio ad-hoc networks, multi-pair primary networks, isolated node probability, link connectivity, path connectivity

## Abstract

In this paper, we study the connectivity of cognitive radio ad-hoc networks (CRAHNs) where primary users (PUs) and secondary users (SUs) are randomly distributed in a given area following a homogeneous Poisson process. Moreover, for the sake of more realistic CRAHNs, contrary to previous works in the literature, we consider the case that primary network is comprised of multiple communication pairs which are spatial-temporal distributed in the network area. We also take into consideration the differences in transmission range and interference range of both PUs and SUs. The connectivity of such CRAHN is studied from three viewpoints. First, we mathematically analyze the probability of isolated secondary transmitter and secondary receiver. Second, we derive the approximation expression of the link probability between two adjacent SUs. Third, we investigate the path connectivity between two arbitrary SUs by using the simulation analysis approach. The correctness of our mathematical expressions is confirmed by comparing analytical results with simulation results. The results in this paper provide insights into how multiple communication pairs in primary network affect the connectivity of secondary network, which can be useful guidelines for the design of CRAHNs.

## 1. Introduction

In recent years, technological advances together with the demand for efficient and flexible networks have led to the development of wireless ad-hoc networks (AHNs). In such networks, mobile devices can communicate with each other in a peer-to-peer fashion with no need for any base station or pre-existing network infrastructure. AHNs have been mostly limited in their operations in the 900 MHz and the 2.4 GHz industrial, scientific, and medical (ISM) bands. With the fast increase in the number of wireless devices, these frequency bands are getting congested. At the same time, many other licensed frequency bands allocated through static polices are used only in bounded geographical area or over a specific period of time. The Federal Communication Commission (FCC) estimated that the average utilization of licensed frequency band varies between 15–18% [1]. To address the critical problem of spectrum scarcity, FCC has recently approved the use of unlicensed devices in licensed bands. This policy has encouraged the development of cognitive radio ad-hoc networks (CRAHNs) [2,3] where unlicensed users (or Secondary Users—SUs) can coexist with licensed users (or Primary Users—PUs) if unlicensed users use licensed frequency bands opportunistically in a dynamic and non-interfering manner to improve spectrum usage efficiency.

Connectivity is an important property of ad-hoc wireless networks. It is one of the main factors that influence the network performance. Connectivity in traditional wireless AHNs has been intensively studied in the literature. The authors in [4] investigated the probability that entire ad-hoc network is connected with the assumption that wireless nodes have fixed circular transmission range and are distributed in disk area. To study connectivity-related properties of AHNs in more accurate models of wireless channel, the influences of log-normal shadowing fading [5] and Rayleigh fading [6] on the connectivity of AHNs were considered. It was shown that the presence of fading could result in an improvement of overall connectivity. The connectivity of wireless AHNs where nodes are equipped with directional antennas was presented in [7,8,9,10]. In [7], the study was performed by using accurate, analytical antenna models, i.e., Uniform Linear Array (ULA) antenna and Uniform Circular Array (UCA) antenna. Using the simulation analysis approach, the authors showed that simple randomized beamforming significantly improved the connectivity among wireless nodes compared with networks with omnidirectional antenna employing the same power. The analytical model for evaluating the combined impact of beamforming formed by ULA and UCA antennas, together with shadowing, on the network connectivity was given in [8]. Moreover, both randomized beamforming and center-directed beamforming schemes were evaluated. The results indicated that beamforming improved the local and the overall connectivity in the case of moderate shadowing. Multi-hop connectivity of AHNs with antenna model given by cardioid function was mathematically investigated in [9]. Comparisons of the influences of various antenna models on the network connectivity of wireless AHNs were shown in [10].

Today, the connectivity of CRAHN has raised increasing awareness of researchers. Investigating on the connectivity of CRAHNs is a challenging issue compared with that of AHNs due to the influence of varying spectrum availability caused by the random locations and activeness of PUs. The reachability between two SUs depends not only on the distance between them but also on the availability of communication channels. Specifically, the communication links among SUs in secondary network are time-space varying due to the dynamic of spectrum opportunities. In [11], the authors analyzed the impact of primary users on the secondary network connectivity by using the second smallest Laplacian eigenvalue. The percolation-based connectivity of large-scale ad-hoc heterogeneous wireless networks was addressed in [12]. The authors introduced the concept of connectivity region, which was defined as a set of density pairs (i.e., the density of secondary users and the density of primary transmitters) under which the secondary network is connected, i.e., there is at least one path between every pair of nodes. However, in many wireless multi-hop network applications, it may not be necessary to require every node to be connected to every other node. Based on the basics of random geometric graph and probability theory [13], the closed-form formula of relation between connectivity and density of PU, density of SUs, and transmission radius of SU was given in [14]. Using aggregate interference model, the authors in [15] investigated the local connectivity of CRAHNs, i.e., node degree and the probability of node isolation. Analysis of connectivity of CRAHNs in log-normal shadow fading environment was given in [16]. The results show that high fading variance helps to remarkably improve the connectivity and the impact of shadow fading on wireless connection probability dominates that of PU’s average active rate. In [17], the authors investigated the probability of node isolation of SU in CRAHNs where both PUs and SUs are equipped with the approximated directional antenna model called sector antenna. It was shown that the probability of node isolation is improved compared with conventional CRAHNs using omnidirectional antennas due to the reduced PU-to-SU interference and the extended transmission range. A modeling framework for evaluating connectivity in CRAHNs where PUs are equipped with omnidirectional antennas while SUs are equipped with directional antennas, i.e., ULA and UCA antennas, was shown in [18]. It was concluded that UCA is the most suitable antenna for CRAHNs and the optimal number of elements at which the highest connectivity can be achieved is different for each type of directional antenna. The connectivity of hybrid CRAHNs in which each SUs can dynamically select overlay cognitive radio or underlay cognitive radio paradigms to access licensed spectrum was studied in [19]. Simulation results showed that by using the hybrid underlay/overlay transmission mode at the SUs, the path probability increased compared with the case of using only underlay mode or overlay mode.

We should note that all aforementioned works consider CRAHNs where PUs were not clearly classified as transmitters or receivers and the differences in transmission range and interference range were not mentioned. In addition, the primary networks comprising of multiple pairs of primary transmitters and receivers was not considered. Motivated by these issues, in this paper we mathematically study the influence of multiple communication pairs of primary network on the connectivity of secondary network. The following is a summary of the main contributions in this paper.

1. In contrast to previous works in the literature which analyzed the connectivity of secondary network without explicitly specifying the transmitting and receiving roles of PUs in communication process, we consider more practical CRAHN where primary network comprises of multiple spatial-temporal one-hop communication pairs of primary transmitter—primary receiver such as in licensed cellular networks, and then analyze the connectivity of secondary network from this perspective.
2. We derive the mathematical models of the isolation probability of SU and the link probability of two adjacent SUs, taking into account the differences in transmission range and interference range of both PUs and SUs. The path connection between two arbitrary SUs is also studied.
3. We show that network size and the number of PU pairs determine the minimum value of SU’s isolation probability and the maximum value of path connectivity between two arbitrary SUs. Moreover, the distance between two adjacent nodes insignificantly affects the link probability between them.

The rest of this paper is organized as follows. In Section 2, we describe the network models of primary network and secondary network. In Section 3, we present the analysis of the connectivity of CRAHNs where multiple one-hop communication pairs of primary transmitter–primary receiver are spatial-temporal distributed. Moreover, the difference in transmission range and interference range of both PUs and SUs is also taken into consideration. In Section 4, numerical results are provided and discussed. Analysis results are confirmed by simulation results to verify the derived mathematical models. Finally, Section 5 concludes the paper.

## 2. System Model

### 2.1. Spatial Node Distribution

**Figure 1:**
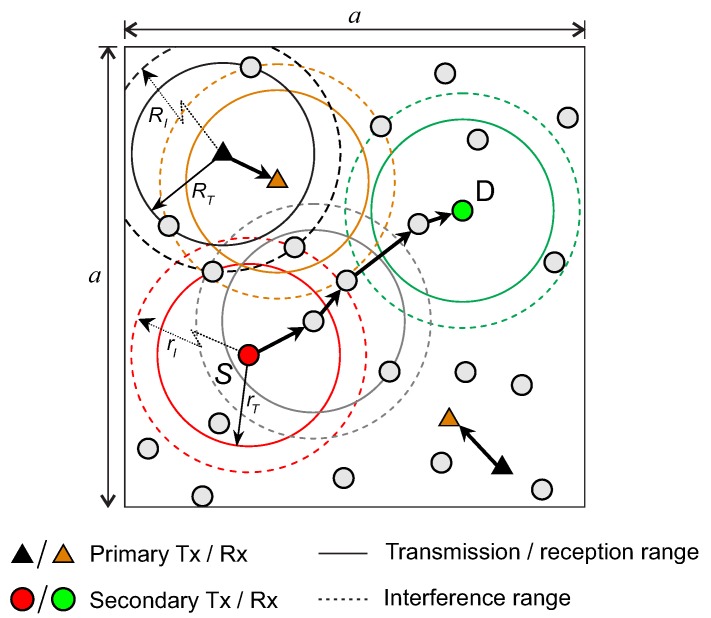
The system model of the considered cognitive radio ad-hoc networks (CRAHNs) with multiple one-hop-communication primary networks and the difference in transmission range and interference range.

We consider CRAHNs where PUs and SUs share a licensed spectrum band and are distributed according to homogeneous Poisson point process [20] in a finite two-dimensional Euclidean network area a×a with density $\rho =N/a^{2}$ nodes per unit area as shown in Figure 1. The homogeneous Poisson point process is defined by the following property.

The number of nodes *q* in a specific finite subarea *A*, e.g., the coverage area of node, follows a Poisson distribution, i.e.,

(1) $$P\left(q\;\mathrm{nodes}\;\mathrm{in}\;A\right)=P\left(Q=q\right)=\frac{\eta^{q}}{q!}e^{-\eta },$$

with an expected value E[Q]=η=ρA.

### 2.2. Primary Network

The primary network is modeled as a random graph where primary transmitters and primary receivers are distributed according to two-dimensional Poisson point process of density $\rho_{P}=N_{P}/a^{2}$. Let the communication of each primary transmitter with primary receiver on licensed spectrum band be associated with an independent and identical distributed ON-OFF state where the number of licensed spectrum occupation per time unit follows Poisson distribution with average active rate $\lambda_{P}$.

(2) $$P\left(X=x\right)=\frac{\lambda_{P}^{x}}{x!}e^{-\lambda }.$$

The ON state represents the interval in which the primary transmitter is active and the OFF state depicts the interval in which the primary transmitter is inactive. Similar to [12,14], we assume that all primary transmitters use the same transmission power and the transmitted signals undergo isotropic path losses with transmission ranges $R_{T}$. Each primary receiver is uniformly distributed within transmission range $R_{T}$ of the primary transmitter. A primary receiver is active or inactive if its associated primary transmitter is active or inactive, respectively. According to the displacement theorem [20], it can be concluded that the primary receivers form a two-dimensional Poisson point process with density $\rho_{P}$.

### 2.3. Secondary Network

The secondary network is also modeled as a random graph where secondary users, acting as both secondary transmitters and secondary receivers, are distributed according to two-dimensional Poisson point process of density $\rho_{S}=N_{S}/a^{2}$. Without having the impact of primary network, secondary network can be considered as AHN. However, in temporal-spatial spectrum agility of CRAHNs as considered in this paper, the wireless communication link between two secondary users is available if there exists spectrum opportunity for them to communicate. All secondary users have equal transmission ranges, denoted by $r_{T}$.

Due to the coexistence of primary network and secondary network in CRAHNs, there exists a spectrum opportunity from the secondary transmitter SU${}_{T}$ to the secondary receiver SU${}_{R}$ if the two following conditions are fulfilled. (1) the transmission from SU${}_{T}$ does not interfere with any active primary receivers in a circular region with radius of $r_{I}$ and (2) the reception of SU${}_{R}$ is not affected by any active primary transmitters in a circular region with radius of $R_{I}$. The radius $r_{I}$ refers to interference range of the secondary users which depends on the transmission power of SU${}_{T}$ and the interference tolerance of the primary receiver, whereas the radius $R_{I}$ refers to the interference range of primary users which depends on the transmission power of primary transmitter and the interference tolerance of SU${}_{R}$. Generally, the transmission range $R_{P}$ of primary users in smaller than the interference range $R_{I}$. In other words, under the signal propagation and interference model, we have $R_{T}=\gamma R_{I}$, (0<γ<1). A similar relationship holds for $r_{T}$ and $r_{I}$. We will use $R_{T}$, $R_{I}$, $r_{T}$, and $r_{I}$ to analyze the following connectivity related properties in CRAHNs. Without loss of generality, we also assume that $R_{T}=r_{T}$ and $R_{I}=r_{I}$ as in [12].

## 3. Connectivity Analysis

Using the system model in Section 2, we consider both the local connectivity (isolation probability of a SU, link connectivity between two adjacent SUs) and the overall connectivity (path connectivity between two SUs) of secondary network. Contrary to previous work [14,15,17], for the shake of more realistic CRAHNs, we take into account the coexistence of multiple one-hop communication pairs of PUs in primary network and the differences in the transmission range and interference range of both PUs and SUs.

### 3.1. Probability of Isolated Secondary User

In this subsection, we study the probability of isolated SU, i.e., the probability that a specific SU does not have available licensed frequency bands and any neighboring nodes to communicate with. Since every SU in secondary network can be either transmitter or receiver, the probability of isolated secondary user can be classified as the probability of isolated secondary transmitters and the probability of isolated secondary receivers.

**Figure 2:**
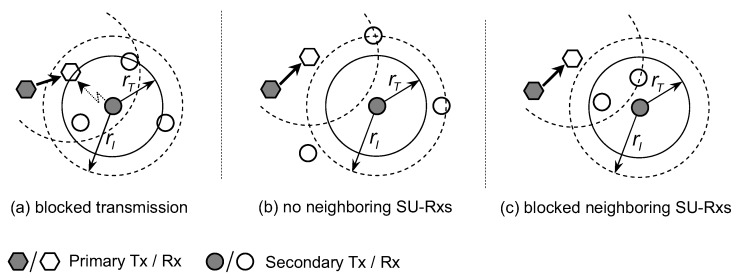
Isolation of secondary transmitter in CRAHNs due to (**a**) blocked transmission, (**b**) no neighboring SU-Rxs, and (**c**) blocked neighboring SU-Rxs, respectively.

From the perspective of secondary transmitter, it is considered as an isolated node if one of the following cases happens:

(i) There is at least one active primary receiver inside its interference range (refer to Figure 2a).

According to (1), the probability that there are *m* primary receivers within the interference area $\pi {r_{I}}^{2}$ of secondary transmitter is given by

(3) $$P_{M}^{PU}\left(M_{PU}=m\right)=\frac{{\left(\pi {r_{I}}^{2}\rho_{P}\right)}^{m}}{m!}e^{-\pi {r_{I}}^{2}\rho_{P}}.$$

where the average number of primary receivers in the transmission area is $\eta_{PU}=\pi {r_{I}}^{2}\rho_{P}$ and $\rho_{P}$ is the density of primary receiver.

In order to have spectrum opportunity for secondary user, it is required that all these *m* primary users are not active. By assigning x=0 in (2), we obtain the probability that a primary user is in OFF state as

(4) $$P_{X}^{PU}\left(X=0\right)=e^{-\lambda_{P}}.$$

Since the operations of primary receivers are mutually independent, the probability that all these *m* primary users are inactive is

(5) $$P_{X}^{PU}\left(X=0,M=m\right)={\left(P_{X}^{PU}\left(X=0\right)\right)}^{m}=e^{-m\lambda_{P}}.$$

Therefore, the probability that no active primary receiver within the interference area of secondary transmitter is obtained by multiplying two probabilities given in (3) and (5), i.e.,

(6) $$\Phi =\sum_{m=0}^{N_{P}}\frac{{\left(\pi {r_{I}}^{2}\rho_{P}\right)}^{m}}{m!}e^{-\pi {r_{I}}^{2}\rho_{P}}e^{-m\lambda_{P}}.$$

and the probability that there is at least one active primary receiver inside the interference range of secondary transmitter is given by

(7) $$P_{tx-block}=1-\Phi =1-\sum_{m=0}^{N_{P}}\frac{{\left(\pi {r_{I}}^{2}\rho_{P}\right)}^{m}}{m!}e^{-\pi {r_{I}}^{2}\rho_{P}}e^{-m\lambda_{P}}.$$

(ii) There are no active primary receivers inside its interference range but there are also no secondary receivers inside its transmission range (refer to Figure 2b).

From (1), the probability that there are no other SUs inside the transmission range of a specific SU is calculated as

(8) $$P_{M}^{SU}\left(M_{SU}=0\right)=e^{-\pi {r_{T}}^{2}\rho_{S}}.$$

Then, by combining (6) with (8), the probability of the event that a secondary transmitter has transmission opportunity but has no secondary receivers is

(9) $$P_{no-SU}=\left(\sum_{m=0}^{N_{P}}\frac{{\left(\pi {r_{I}}^{2}\rho_{P}\right)}^{m}}{m!}e^{-\pi {r_{I}}^{2}\rho_{P}}e^{-m\lambda_{P}}\right)e^{-\pi {r_{T}}^{2}\rho_{S}}.$$

(iii) There are no active primary receivers inside its interference range $r_{I}$, there also exists secondary receivers inside its transmission range $r_{T}$ but these secondary receivers are blocked by active primary transmitters (refer to Figure 2c).

Since this event relates to several spatial dependence problems, deriving the mathematical expression is very challenging. Thus, the probability of isolated secondary transmitter given in (10) is approximately calculated as the summation of (7) and (9). We will prove in Section 4 that this probability is negligible in moderate and large network areas.

(10) $$\begin{array}{ll} P_{iso}^{tx} & \approx P_{tx-block}+P_{no-SU}\\ & \approx 1-\sum_{m=0}^{N_{P}}\frac{{\left(\pi {r_{I}}^{2}\rho_{P}\right)}^{m}}{m!}e^{-\pi {r_{I}}^{2}\rho_{P}}e^{-m\lambda_{P}}+\left(\sum_{m=0}^{N_{P}}\frac{{\left(\pi {r_{I}}^{2}\rho_{P}\right)}^{m}}{m!}e^{-\pi {r_{I}}^{2}\rho_{P}}e^{-m\lambda_{P}}\right)e^{-\pi {r_{T}}^{2}\rho_{S}}\\ & \approx 1-\left(1-e^{-\pi {r_{T}}^{2}\rho_{S}}\right)\sum_{m=0}^{N_{P}}\frac{{\left(\pi {r_{I}}^{2}\rho_{P}\right)}^{m}}{m!}e^{-\pi {r_{I}}^{2}\rho_{P}}e^{-m\lambda_{P}}. \end{array}$$

For secondary receiver, the node is considered as isolated node if (i) *it is within the interference range of at least one active primary transmitter* or (ii) *it is not interfered by any active primary transmitter but it is not in the transmission range of any secondary transmitters*, or (iii) *it is not interfered by any active primary transmitter but all its neighboring secondary transmitters are blocked*. Similarly, the probability of isolated secondary receiver can be approximately expressed as

(11) $$P_{iso}^{rx}\approx 1-\left(1-e^{-\pi {r_{T}}^{2}\rho_{S}}\right)\sum_{m=0}^{N_{P}}\frac{{\left(\pi {R_{I}}^{2}\rho_{P}\right)}^{m}}{m!}e^{-\pi {R_{I}}^{2}\rho_{P}}e^{-m\lambda_{P}}.$$

### 3.2. Link Connectivity between Two Adjacent Secondary Users

**Figure 3:**
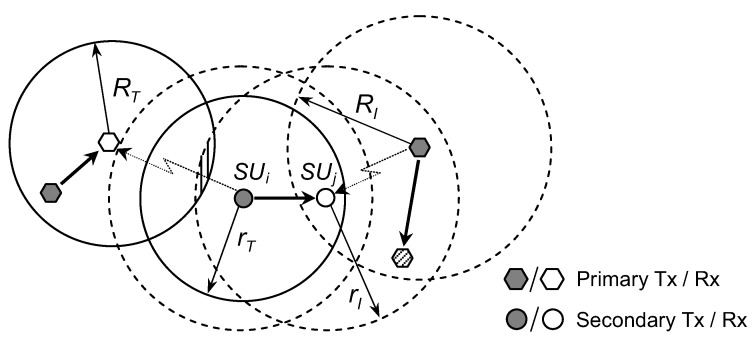
Link connectivity between two adjacent nodes in CRAHNs.

Figure 3 shows the communication between a secondary transmitter SU${}_{i}$ and a secondary receiver SU${}_{j}$. SU${}_{i}$ and SU${}_{j}$ are called adjacent nodes because SU${}_{j}$ is in the coverage area of SU${}_{i}$. The transmission range of SU${}_{i}$ is $r_{T}$. It is easy to see that from viewpoint of SU${}_{j}$, its required protection range is equal to the interference range of primary transmitter, $R_{I}$. Due to spatial-dependent probability, obtaining the exact mathematical expression of link connectivity between two adjacent SUs is sophisticated. Therefore, we derive closed-formed approximate expressions in the following parts and prove in Section 4 that the analysis results are not much different from the simulation results and can reflect the average value of link probability.

From the viewpoint of secondary transmitter, it is allowed to transmit packet if there are no active primary receivers inside its interference area $\pi {r_{I}}^{2}$. According to (6), the probability that secondary transmitter SU${}_{i}$ is allowed to transmit is

(12) $$\Phi_{SU_{i}}=\sum_{m=0}^{N_{P}}\frac{{\left(\pi {r_{I}}^{2}\rho_{P}\right)}^{m}}{m!}e^{-\pi {r_{I}}^{2}\rho_{P}}e^{-m\lambda_{P}}.$$

Similar to the case of secondary transmitter, the probability that secondary receiver SU${}_{j}$ can receive packet if it does not stay within the interference area $\pi {R_{I}}^{2}$ of any active primary transmitters, i.e.,

(13) $$\Theta_{SU_{j}}=\sum_{n=0}^{N_{P}}\frac{{\left(\pi {R_{I}}^{2}\rho_{P}\right)}^{n}}{n!}e^{-\pi {R_{I}}^{2}\rho_{P}}e^{-n\lambda_{P}}.$$

A wireless communication link between two adjacent nodes, i.e., the secondary transmitter SU${}_{i}$ and the secondary receiver SU${}_{j}$, exists if and only if SU${}_{i}$ and SU${}_{j}$ does not interference with and is not interfered by any active primary receivers and primary transmitters, respectively. Therefore, we can express the link probability between them as

(14) $$P_{link}\approx \Phi_{SU_{i}}\Theta_{SU_{j}}=\sum_{m=0}^{N_{P}}\sum_{n=0}^{N_{P}}\frac{{\left(\pi r_{I}^{2}\rho_{P}\right)}^{m}}{m!}\frac{{\left(\pi R_{I}^{2}\rho_{P}\right)}^{n}}{n!}e^{-\pi \left(r_{I}^{2}+R_{I}^{2}\right)\rho_{P}}e^{-\left(m+n\right)\lambda_{P}}.$$

### 3.3. Path Probability between Two Arbitrary Secondary Users

**Figure 4:**
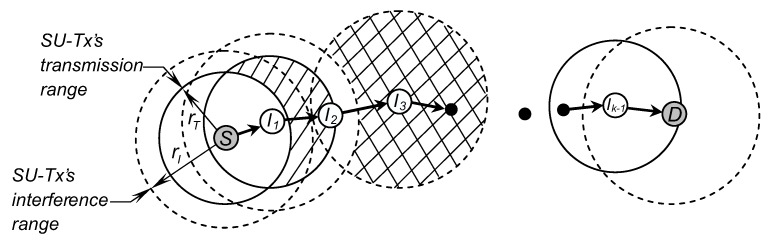
Multi-hop connectivity between two secondary users (SUs) in CRAHNs.

From the link connectivity between two adjacent SUs presented in Section 3.2, we now consider the path connectivity between two arbitrary SUs, i.e., source node (S) and destination node (D), in CRAHNs. As depicted in Figure 4, the transmission on a *k*-hop path consists of the following phases: (i) source node S sends packet to intermediate node I${}_{1}$, (ii) I${}_{1}$ successfully receives the packets from S, (iii) I${}_{1}$ now acts as a transmitter to forward packets to next node I${}_{2}$, and (iv) I${}_{2}$ receives the packets and forwards to its next node. The process repeats until packets reach destination node D.

The mathematical analysis of hop count distribution between two arbitrary nodes still remains an open research issue because deriving the analytical models of hop count between two arbitrary SUs and the spatial dependent connection of wireless links on the path is very challenging. Hence, the path connectivity between two random nodes is often studied by using simulation [5,8]. Similar to those works, in this paper, we will study the path connectivity by using the simulation analysis approach. Path establishment among SUs in secondary network is based on a greedy forwarding (GF) routing algorithm [21] that operates according to the following rules: (1) every node selects the neighbor which is closest to the destination as the forwarding node. (2) if a node cannot find a next-hop neighbor that is nearer to the destination than itself, the path from it to the destination does not exist. Because of the advantages such as distributed feature and simple routing algorithm with low control overhead, GF routing algorithm is widely used in wireless multihop networks. The simulation analysis of path connectivity between two random secondary users in CRAHNs with GF routing algorithm was also used in our previous works [22,23].

## 4. Numerical Results

In this section, we present the numerical results of the probability of isolated SU, the link probability between two adjacent SUs, and the path connectivity between two arbitrary SUs. We verify the mathematical models of isolation probability and link probability given in Section 3.1 and Section 3.2 by comparing analysis results with simulation results. Monte Carlo simulations are conducted by using MATLAB on a computer workstation. Simulation results are calculated by averaging 50,000 network topologies. The simulation time for each value in the result graphs depends on the values of network parameters such as the number of SUs and network size used in each simulation scenario.

**Figure 5:**
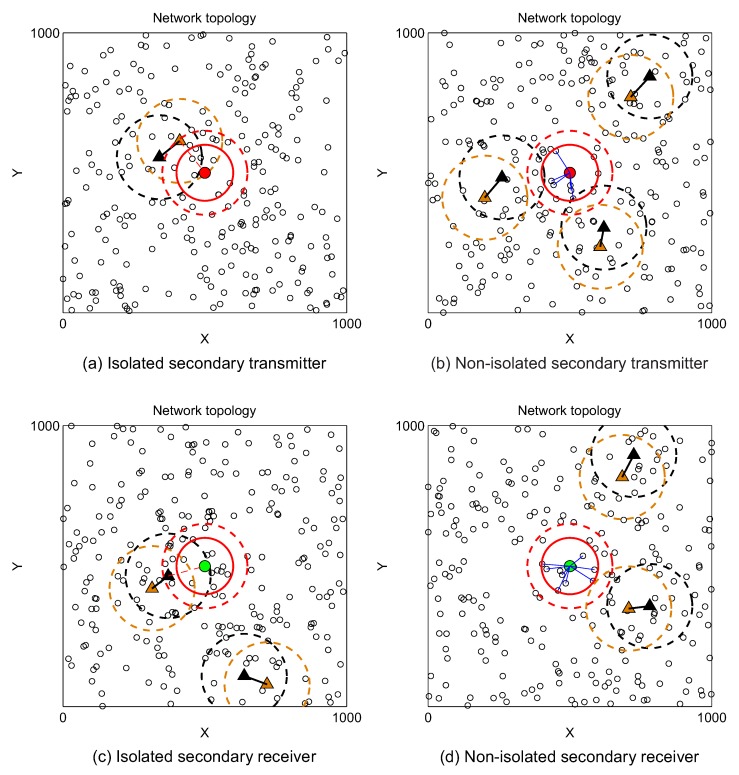
Illustration of isolation possibility of secondary transmitter and receiver. *a* = 1000 m, $N_{S}$ = 250, $N_{P}$ = 5, $\lambda_{P}$ = 0.5, $R_{T}$ = $r_{T}$ = 100 m, $R_{I}$ = $r_{I}$ = 150 m.

Figure 5 illustrates the cases where secondary transmitter (red circle)/receiver (green circle) are isolated because of being blocked by an active primary receiver (brown triangle)/primary transmitter (black triangle) (refer to Figure 5a,c, respectively) and the cases where they are allowed to transmit/receive data normally (Figure 5b,d, respectively).

**Figure 6:**
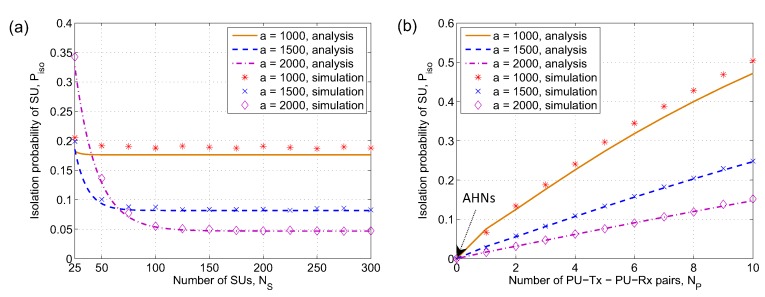
Probability of isolated SU versus (**a**) the number of secondary users, $N_{S}$, and (**b**) the number of PU-Tx-PU-Rx pairs, $N_{P}$, with different network sizes, *a*. $\lambda_{P}$ = 0.3, $R_{T}$ = $r_{T}$ = 250 m, $R_{I}$ = $r_{I}$ = 280 m; $N_{P}$ = 3 for (**a**) and $N_{S}$ = 300 for (**b**).

To quantitatively study the isolation probability of SUs and verify the correctness of (10) and (11) in Section 3, we plot the analysis results and simulation results of isolation probability of SUs for $\lambda_{P}$ = 0.3, $R_{T}$ = $r_{T}$ = 250 m, $R_{I}$ = $r_{I}$ = 280 m. The probability of isolated SU versus the number of SUs is shown in Figure 6a. Network size, *a*, is varied from 1000 m to 2000 m. From Figure 6a, we can see an interesting feature, i.e., the isolation probability of SU, $P_{iso}$, greatly depends on network size, *a*, and the number of SUs, $N_{S}$. Specifically, when the number of SUs is very small, i.e., $N_{S}$ = 25, the isolation probabilities of SUs in the cases of *a* = 1000 m and *a* = 1500 m are very similar ($P_{iso}$ = 0.182 and $P_{iso}$ = 0.185, respectively). However, in the case of *a* = 2000 m, the isolation probability of SU is much higher ($P_{iso}$ = 0.362) because SU density is exponentially decreased with respect to the increase in network size. As the number of SUs in the network area gets higher, the isolation probability of SU remarkably decreases until it reaches the minimum values of 0.082 (−55.68%) when $N_{S}$ = 76, *a* = 1500 m, and 0.046 (−87.29%).

The isolation probability of SU as a function of the number of PU-Tx-PU-Rx pairs is depicted in Figure 6b. As observed in Figure 6b, when there are no primary users, i.e., $N_{P}$ = 0 and the CRAHNs becomes AHNs, every SU can always communicate with other SUs, i.e., $P_{iso}$ = 0, in all settings of network sizes. However, it should be noted that having a greater number of PU-Tx-PU-Rx pairs results in the increase in the isolation probability of SU. Especially, $P_{iso}$ grows rapidly when network size is small, i.e., *a* = 1000 m. More particularly, as $N_{P}$ increases from 1 to 10, $P_{iso}$ increases from 0.017 to 0.148, from 0.031 to 0.247, and from 0.076 to 0.472 for the cases of network sizes *a* = 1000 m, 1500 m, and 2000 m, respectively. In both Figure 6a,b, the analysis results closely match with the simulation results when network size is large (*a* = 1500 m and *a* = 2000 m) because the probability that all neighbors of a SU are blocked by active PUs is alleviated. In the case of small network size, there are gaps between the analysis results and simulation results. However, the gaps are not very big and from the perspective of low complexity of closed-form mathematical model, this tolerance is acceptable.

**Figure 7:**
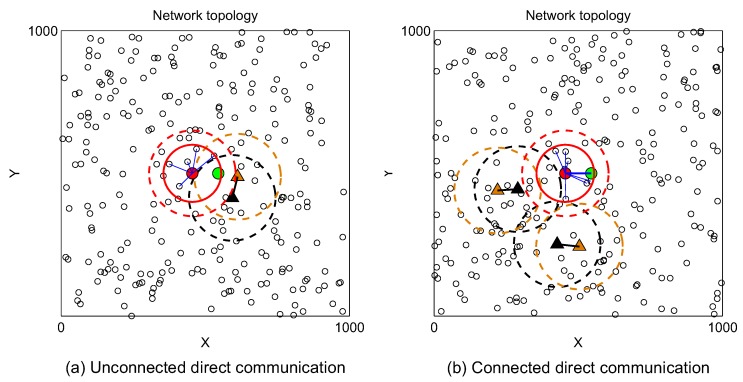
Illustration of direct communication between two SUs. *a* = 1000 m, $N_{S}$ = 250, $N_{P}$ = 5, $\lambda_{P}$ = 0.5, $R_{T}$ = $r_{T}$ = 100 m, $R_{I}$ = $r_{I}$ = 150 m.

Next, we will study the possibility of having a direct communication link between two adjacent SUs in CRAHNs. Figure 7a illustrates the case that direct communication link cannot be established because the secondary receiver (green circle) is blocked by an active primary transmitter (black triangle). On the other hand, there exists direct communication link in Figure 7b because secondary transmitter (red circle) does not interfere with any active primary receivers (brown triangles) and secondary receiver is not interfered by any active primary transmitters.

**Figure 8:**
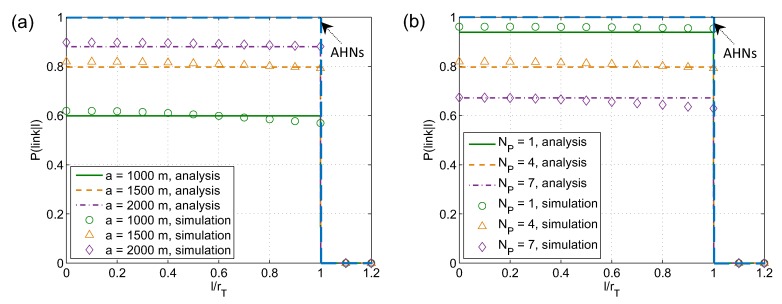
Link connectivity between two nodes versus the relative distance between them for different (**a**) network size *a* and (**b**) the number of PU-Tx-PU-Rx pairs. $N_{S}$ = 300, $\lambda_{P}$ = 0.3, $R_{T}$ = $r_{T}$ = 250 m, $R_{I}$ = $r_{I}$ = 280 m; $N_{P}$ = 4 for (**a**) and *a* = 1500 m for (**b**).

Figure 8 shows the probability of having a link between two SUs versus the relative distance $l/r_{T}$ for different values of network size, *a*, and the number of PU-Tx-PU-Rx pairs, $N_{P}$. The analysis results obtained from (14) in Section 3.2 are compared with simulation results. Overall, this mathematical model can reflect the average value of link probability. The simulation result of link probability is higher than the average value when $l/r_{T}$ is small and reduces as $l/r_{T}$ increases. This feature can be explained as follows. When the distance between SU-Tx (SU${}_{i}$) and SU-Rx (SU${}_{j}$), *l*, is much smaller than transmission range, $r_{T}$, the mutual impacts on SU${}_{j}$ of the primary transmitters of the primary receivers interfered by SU${}_{i}$, i.e., PU-Txs(PU-Rxs(SU${}_{i}$))) ⇒ SU${}_{j}$, and vice versa are considerable, leading to higher possibility that SU${}_{i}$ and SU${}_{j}$ are blocked. As the distance *l* gets higher, this effect is relieved. In addition, as observed from Figure 8, similar to AHNs, it is obvious that when the distance between secondary transmitter and secondary receiver is larger than transmission range (or $l/r_{T}>1$) they cannot communicate directly. We can also see that link probability is not linearly proportional to both *a* and $N_{P}$. Especially, varying $N_{P}$ results in more significant influence than varying *a*.

**Figure 9:**
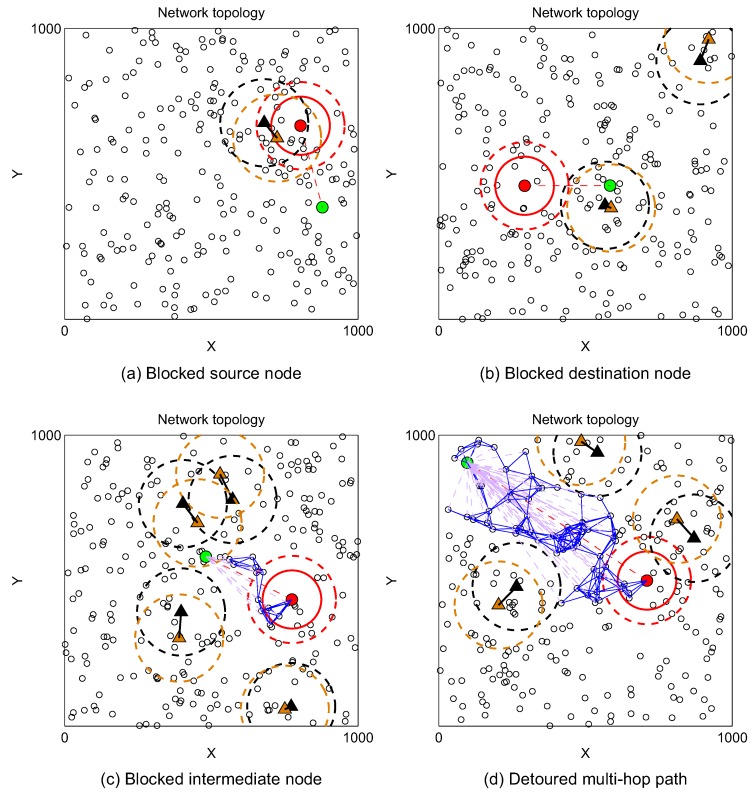
Illustration of multi-hop communication between two SUs in CRAHNs. *a* = 1000 m, $N_{S}$ = 250, $N_{P}$ = 5, $\lambda_{P}$ = 0.5, $R_{T}$ = $r_{T}$ = 250 m, $R_{I}$ = $r_{I}$ = 280 m.

We are now interested in examining the path connectivity between two arbitrary nodes. Figure 9a–c present the cases that source SU (red circle) cannot establish communication path with destination SU (green circle) because source node, destination node, and intermediate node are blocked by active PUs, respectively. Figure 9d illustrates the case where there exits routing paths from source SU to destination SU. However, due to the coexistence of active PUs, the routing paths have to detour.

**Figure 10:**
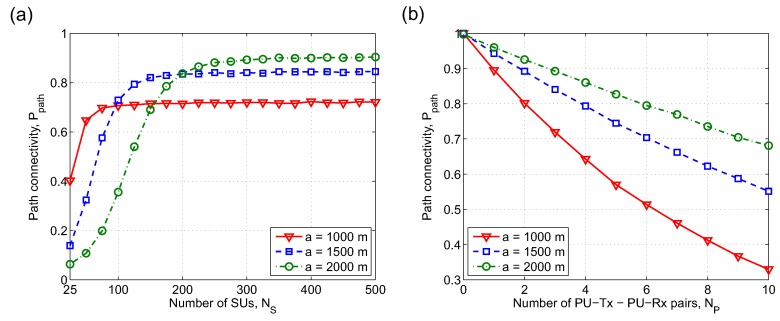
Path connectivity between two arbitrary nodes versus (**a**) the number of SUs, $N_{S}$, and (**b**) the number of PU-Tx-PU-Rx pairs, $N_{P}$, with different network sizes, *a*. $\lambda_{P}$ = 0.3, $R_{T}$ = $r_{T}$ = 250 m, $R_{I}$ = $r_{I}$ = 280 m. $N_{P}$ = 3 for (a) and $N_{S}$ = 300 for (b).

Figure 10a shows the path connectivity versus the number of SUs, $N_{S}$, for different network sizes, *a*. From Figure 10a, we can see that when $N_{S}$≤ 90, path connectivity corresponding to *a* = 1000 m is always higher than those of *a* = 1500 m and 2000 m. This is because when the number of SUs is small, larger network areas result in remarkably low path connectivity between two random SUs due to the following combined effects: (i) lower node density increases the possibility that a SU has no neighbors, and (ii) longer path in terms of hop count makes the path establishment more difficult. As more SUs are added into the networks, path connectivity of each setting of network size *a* increases rapidly, reaches the maximum value and then keeps constant. The maximum value of path connectivity when *a* = 2000 m is $P_{path}$ = 0.89 which is higher than those when *a* = 1500 m ($P_{path}$ = 0.84) and when *a* = 1000 m ($P_{path}$ = 0.71). This is because when SU density is high enough, failures in path establishment mainly come from the influence of PUs, not from the lack of SU neighbors. Thus, with the same settings of $N_{P}$ and $\lambda_{P}$ of primary network, larger network sizes alleviate the impact of PUs, i.e., creating more available area for routing among SUs.

Figure 10b plots the path connectivity, $P_{path}$, versus the number of PU-Tx-PU-Rx pairs, $N_{P}$, for different network sizes *a*. The results in Figure 10b show that path connectivity degrades rapidly as a greater number of PU-Tx-PU-Rx pairs appear in the networks, especially when network size is small. More specifically, in the range of $N_{P}$ from 0 to 10, $P_{path}$ reduces from 0.997 to 0.681 (−31.69%), from 0.999 to 0.552 (−44.74%), and from 1 to 0.329 (−67.10%) corresponding to network sizes *a* = 2000 m, *a* = 1500 m, and *a* = 1000 m, respectively. The reason is that when the number of PU-Tx-PU-Rx pairs increases, the remaining network area for communication among SUs is greatly reduced.

## 5. Conclusions

This paper is motivated by the fact that the connectivity properties of CRAHN should be investigated in the case that primary network is comprised of multiple communication pairs and the transmission range and interference range of both PUs and SUs are different. We have studied the connectivity of CRAHNs in three different viewpoints: the viewpoint of a single node, wireless link, and multi-hop path. More specifically, we mathematically analyze the probability of isolated secondary transmitter and secondary receiver, the link connectivity between two adjacent SUs, and study the path connectivity between two arbitrary SUs by using the simulation approach. The major results observed in this paper can be summarized as follows.

1. For a given network size, the isolation probability of SU, $P_{iso}$, rapidly decreases as the number of SUs goes up to a certain value, then reaches a stable minimum value. However, $P_{iso}$ noticeably increases without reaching a stable value as more number of PU-Tx-PU-Rx pairs in the network.
2. The distance between two adjacent SUs does not noticeably influence the link probability $P_{link}$ of the connection between them. Instead, network size and the number of PU-Tx-PU-Rx pairs determine the maximum value of $P_{link}$.
3. In contrast to the isolation probability of SU, the path probability $P_{path}$ between two random SUs remarkably increases up to a maximum value as the number of SUs is higher. $P_{path}$ continuously decreases with the number of PU-Tx-PU-Rx pairs. It can be observed that the stable minimum value of SU’s isolation probability is inversely proportional to network size *a* while the stable maximum value of path probability is proportional to *a*.

Based on the analysis framework in this paper, further studies of the connectivity of CRAHN with multi-pair primary network for different wireless channel models and antenna models and under the impacts of other factors such as latency, packet congestion can be carried out. 

## References

[1] Federal Communications Commission (2002). Spectrum Policy Task Force, Technical Report. https://transition.fcc.gov/sptf/files/SEWGFinalReport_1.pdf.

[2] Mitola J., Maguire G.Q. (1999). Cognitive radio: Making software radios more personal. IEEE Pers. Commun..

[3] Akyildiz I.F., Lee W.-Y., Chowdhury K.R. (2009). Crahns: Cognitive radio ad hoc networks. Ad Hoc Netw..

[4] Bettstetter C. (2004). On the connectivity of ad hoc networks. Comput. J..

[5] Bettstetter C., Hartmann C. (2005). Connectivity of wireless multihop networks in a shadow fading environment. Wirel. Netw..

[6] Zhou X., Durrani S., Jones H.M. Connectivity of ad hoc networks: Is fading good or bad?. Proceedings of the 2nd International Conference on Signal Processing and Communication Systems (ICSPCS 2008).

[7] Bettstetter C., Hartmann C., Moser C. How does randomized beamforming improve the connectivity of ad hoc networks?. Proceedings of the 2005 IEEE International Conference on Communications (ICC).

[8] Zhou X., Durrani S., Jones H.M. (2009). Connectivity of wireless ad hoc networks with random beamforming. IEEE Trans. Veh. Technol..

[9] Georgious O., Nguyen C. (2015). Multihop connectivity of ad hoc networks with randomly oriented directional antennas. IEEE Wirel. Commun. Lett..

[10] Wang Q., Dai H.-N., Zhao Q. Connectivity of wireless ad hoc networks: Impacts of antenna models. Proceedings of the 2013 International Conference on Parallel and Distributed Computing, Applications and Technologies (PDCAT).

[11] Cuomo F., Abbagnale A., Gregorini A. Impact of primary users on the connectivity of a cognitive radio network. Proceedings of the 9th IFIP Annual Mediterranean Ad Hoc Networking Workshop (Med-Hoc-Net).

[12] Ren W., Zhao Q., Swami A. (2011). Connectivity of heterogeneous wireless networks. IEEE Trans. Inf. Theory.

[13] Haenggi M., Andrews J.G., Baccelli F., Dousse O., Franceschetti M. (2009). Stochastic geometry and random graphs for the analysis and design wireless networks. IEEE J. Sel. Areas Commun..

[14] Liu J., Zhang Q., Zhang Y., Wei Z., Ma S. Connectivity of two nodes in cognitive radio ad hoc networks. Proceedings of the 2013 IEEE Wireless Communications and Networking Conference (WCNC): Networks.

[15] Zhai D., Sheng M., Wang X., Zhang Y. Local connectivity of cognitive radio ad hoc networks. Proceedings of the 2014 IEEE Global Communication Conference (GLOBECOM).

[16] Dung L.T., An B. (2015). Connectivity analysis of cognitive radio ad-hoc networks with shadowing. KSII Trans. Internet Inf. Syst..

[17] Wang Y., Wang Q., Dai H.N., Wang H., Zheng Z., Li J. On Local Connectivity of Cognitive Radio Ad Hoc Networks with Directional Antennas. Proceedings of the 2016 IEEE Internal Conference on Communication System (ICCS).

[18] Dung L.T., An B. (2017). A modeling framework for supporting and evaluating connectivity in cognitive radio ad-hoc networks with beamforming. Wirel. Netw..

[19] Do N.T., Dung L.T., An B., Nam S.-Y. Connectivity of hybrid overlay/underlay cognitive radio ad hoc networks. Proceedings of the 2016 International Conference on Electronics, Information, and Communications (ICEIC).

[20] Kingman J.F.C. (1993). Poisson Processes.

[21] Karp B., Kung H.T. (2000). GPSR: Greed Perimeter Stateless Routing for Wireless Networks. Proceedings of the 6th Annual International Conference on Mobile Computing and Networking (MobiCom ’00).

[22] Dung L.T., Hieu T.D., Choi S.-G. (2016). Simulation modeling and analysis of the hop count distribution in cognitive radio ad-hoc networks with shadow fading. Simul. Model. Pract. Theory.

[23] Dung L.T., Choi S.-G. (2018). Simulation modeling and analysis of the hop count distribution in cognitive radio ad-hoc networks with beamforming. Simul. Model. Pract. Theory.

